# PRRS management by European swine veterinarians: a descriptive insight into practices and profiles

**DOI:** 10.1186/s40813-025-00467-0

**Published:** 2025-11-05

**Authors:** Charlotte Teixeira Costa, Arnaud Lebret, Stéphanie Bougeard, Nicolas Rose

**Affiliations:** 1 Rezoolution, Pig Consulting Services, Noyal-Pontivy, France; 2 ANSES, Epidemiology, Health and Welfare Research Unit, Ploufragan, France

**Keywords:** PRRSV, Swine veterinarians, Evidence-based veterinary medicine, Clustering, Stabilization programme, European survey, Veterinary profiles

## Abstract

**Background:**

Despite extensive research on porcine reproductive and respiratory syndrome virus (PRRSV), herd-level management strategies vary considerably according to geographical area and depend significantly on many factors. Swine veterinarians play a key role in PRRS management; however, their practices, perceptions, and decision-making processes remain insufficiently described. This study aimed to characterize distinct profiles of European swine veterinarians and explore how these profiles relate to their experience with PRRSV stabilization programmes.

**Results:**

A cross-sectional online survey targeting swine veterinarians in 22 European countries was conducted between 2023 and 2024 (600 sent questionnaires). The questionnaire covered sociodemographic data, professional interactions, engagement with evidence-based veterinary medicine (EBVM), PRRS management strategies, and stabilization programme experience. Multivariate analysis and hierarchical clustering on principal components were used to identify practitioner profiles and explore their associations with PRRS control-related variables. Among 108 respondents, five thematic blocks revealed a significant heterogeneity in veterinary profiles. Three distinct clusters were identified per thematic which were related to two PRRS context profiles and four stabilization outcome groups (“total success”, “partial success due to farmer limitations”, “partial success due to technical limitations” and “never implemented”). Successful implementation of PRRSV stabilization programmes was more frequently associated with veterinarians with long-lasting experience, open to EBVM, well-integrated in collaborative networks, and who had reliable access to diagnostic tools and vaccines.

**Conclusion:**

This study highlights the diversity of European swine veterinarians in their approach to PRRS control, and the profile of veterinarians most likely associated with successful PRRS management. These profiles can help inform more effective training, policy, and communication interventions aimed at improving disease control outcomes in swine production systems.

**Supplementary Information:**

The online version contains supplementary material available at 10.1186/s40813-025-00467-0.

## Background

Porcine reproductive and respiratory syndrome virus (PRRSV) remains one of the most economically significant diseases affecting swine production worldwide. Despite decades of research, its control in the field remains highly variable, and the gap between experimental findings and real-world outcomes continues to challenge veterinarians and producers [1]. As key players in pork production chain, swine veterinarians are not only expected to manage clinical cases, but also to act as advisors on herd-level strategies, health policies, and biosecurity protocols [2].

Evidence-based veterinary medicine (EBVM), defined as the conscientious integration of the best available scientific evidence with clinical expertise and patient needs, is increasingly promoted as a tool to improve decision-making and harmonize practices [3]. However, applying EBVM principles in swine medicine is often hindered by structural and contextual limitations. Barriers to knowledge translation − including time constraints, access to data, communication between stakeholders, and practical relevance of scientific evidence − have been documented among swine veterinarians [4, 5]. These constraints are particularly salient in managing complex infectious diseases such as PRRS, where clinical signs, transmission dynamics, and vaccine efficacy vary considerably. Understanding how veterinarians differ in their approach to PRRS control is crucial for designing targeted education, communication, and policy strategies. Indeed, EBVM-based handling of PRRSV management procedures in pig farms typically combines rigorous diagnosis, vaccination strategies, biosecurity measures, and structured herd-stabilization or elimination programmes. Field studies have shown that integrating laboratory diagnostics to monitor virus circulation in weaned piglets, mass vaccination of breeding herds, strict internal and external biosecurity (e.g., herd closure, unidirectional animal and human flows, reduced cross-fostering), and monitoring of key production and health indicators can markedly improve the chances of achieving PRRSV stability or elimination [6, 7]. Similar “closure and rollover” approaches have been successfully implemented for PRRSV elimination in farrow-to-finish herds [8]. The effectiveness of these protocols depends on the consistent application of EBVM principles: choosing interventions based on published evidence, adapting them to local epidemiological conditions, and regularly auditing outcomes to adjust management practices.

Although these challenges are well-recognized, little is known about how they translate into day-to-day decision-making practices across different veterinary profiles. Most existing studies focus on identification of general barriers to EBVM or explore ethical tensions in specific situations (e.g., pain management [9], euthanasia [10]), but few have attempted to systematically describe the diversity of veterinarians’ approaches to PRRS control. Moreover, data from different countries on swine veterinarians’ practices, interactions, and perceptions of available tools remain scarce.

This study addresses this gap by providing a contemporary overview of European swine veterinarians’ practices and perspectives in the context of PRRS management. Indeed, swine veterinarians face substantial challenges in implementing effective PRRS control strategies, including herd stabilization programmes. In this context, we defined “PRRS herd stabilization” as the achievement of a consistent negative PCR status in weaned piglets over a defined period, indicating that the virus is no longer actively circulating within the breeding herd [6, 11].

By identifying descriptive profiles based on sociodemographic characteristics, EBVM engagement, and interaction networks, the study sheds light on the heterogeneity of veterinary profiles and explores how different practitioner types may face distinct challenges and potentially require tailored support in disease control strategies. Thus, this study has two main objectives: first, to identify different profiles of swine veterinarians based on a range of thematic variables; and second, to use these profiles as a framework to identify multiple characteristics related to their attitude towards PRRS management and their level of success regarding PRRS herd stabilization.

## Materials and methods

### Data

They were collected through a cross-sectional survey distributed by email between 2023 and 2024. The survey was distributed to approximately 600 swine veterinarians practicing in 22 European countries through different ways as the French Association for Pig Health (ANSP), the French Interprofessional Pork Council (INAPORC), the European College of Porcine Health Management (ECPHM), the national swine veterinarian’s institutions or colleagues. To be eligible, respondents had to dedicate at least 50% of their professional activity to swine health. The survey aimed to collect detailed information on sociodemographic characteristics, professional interactions, attitudes toward EBVM, PRRS management practices including diagnostics, national or regional regulations, eradication and/or control strategies. To ensure consistency in interpretation, the definition of EBVM was explicitly presented to all participants at the beginning of the EBVM part of the questionnaire. The introductory statement read: “In this survey, EBVM refers to the decisions that combine clinical expertise, the most relevant and best available scientific evidence, and farm-specific context in clinical decision-making”. This step was taken to ensure a shared understanding of the concept across all respondents.

The questionnaire (56 variables, available upon request) was adapted from a previously validated instrument [5] initially focused on EBVM-related aspects and expanded to include the following five thematic blocks: *(i)* sociodemographic characteristics (age, gender, years of experience, employment status, company size, specialization), *(ii)* professional interactions and information exchanges (contacts with pharmaceutical companies and from diagnostic laboratories, other practitioners, farmers, administrative personal), *(iii)* EBVM perception and implementation, *(iv)* PRRS management strategies and practices (diagnosis, context, tools…), and *(v)* experience with PRRS and more specifically PRRS control programmes like stabilization protocols. In this last block, we considered the questions: “Have you ever implemented a PRRSV stabilization programmes?” and, if applicable, “What was your success rate?” For respondents who reported less than full success (< 100%), an additional question was included: “In your opinion, what was missing to achieve 100% success?”, aiming to capture perceived barriers and unmet needs in the implementation process.

### Statistical analysis

**Fig. 1:**
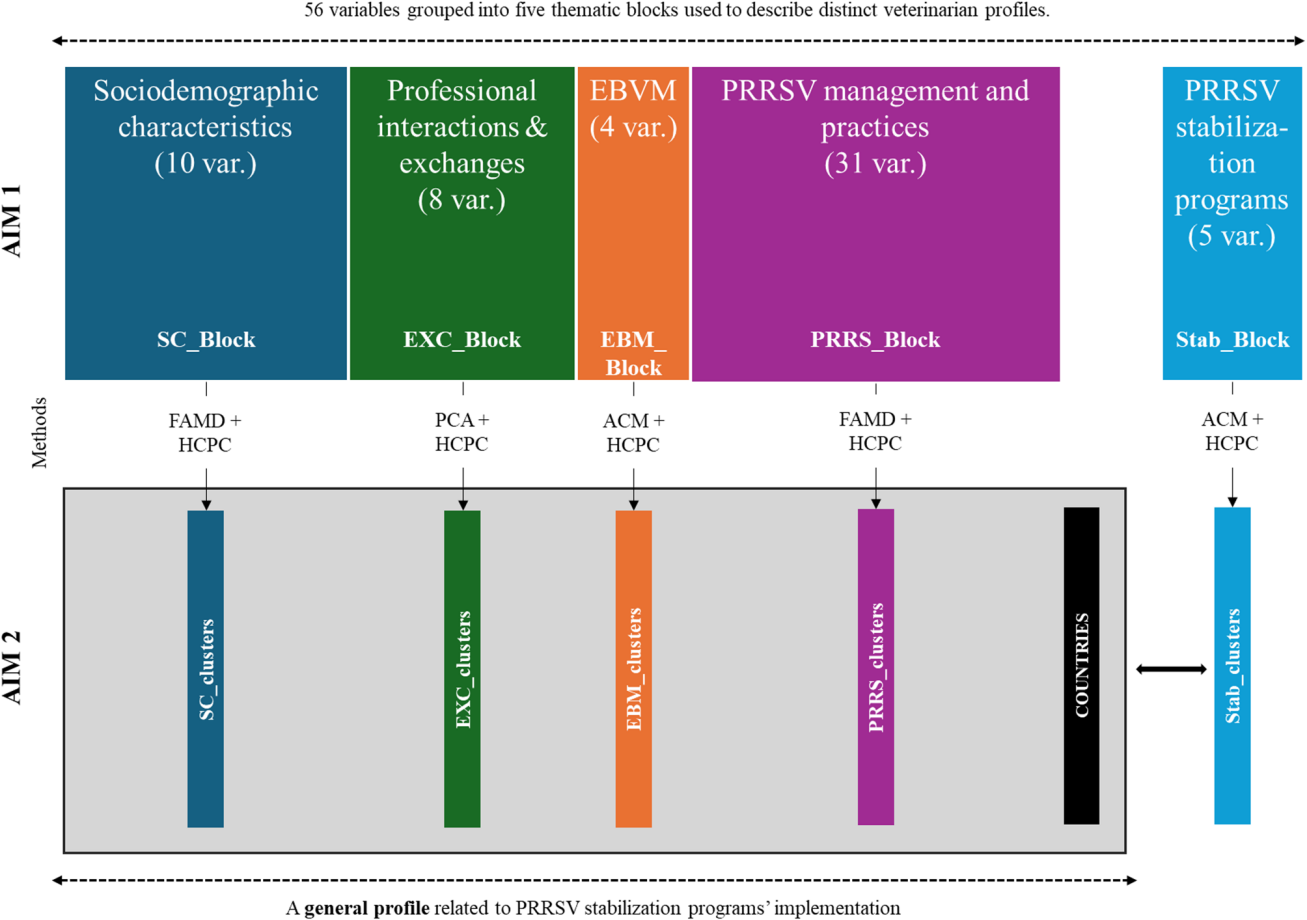
Data and associated statistical workflow. The survey was organized into five analytical blocks: sociodemographic characteristics (SC_Block), professional interactions and exchanges (EXC_Block), evidence-based veterinary medicine perception and practices (EBM_Block), PRRS management practices (PRRS_Block), and experience with PRRSV stabilization programmes (Stab_Block). Each block contained a defined set of variables (resp. 10, 8, 4, 31 and 5 variables). For each block, hierarchical clustering on principal components (HCPC) was applied. The “country” variable was used as a supplementary factor in the final cross-block analysis to account for geographic disparities in responses

The objective of the analysis was to identify and describe distinct profiles of European swine veterinarians based on their practices and attitudes toward PRRS control. The analytical framework used to build these profiles is summarized in Fig. 1, which illustrates the data and the associated statistical workflow. To construct these profiles, a classification approach was applied in order to group veterinarians into homogeneous clusters. Prior to clustering, dimensionality reduction techniques were employed to summarize the information within each thematic block of the questionnaire and to facilitate interpretation of the resulting clusters.

Variables collected in the questionnaire were first categorized as continuous (e.g. age), binary (e.g. yes/no responses), or categorical (e.g. type of practice, country of activity). The detailed list of variables and their characteristics is presented in Appendix 1. Each block of variables was then analysed using an appropriate dimensionality reduction technique: Principal Component Analysis (PCA) was used for blocks composed of continuous variables, Multiple Correspondence Analysis (MCA) for categorical variables, and Factor Analysis of Mixed Data (FAMD) for mixed datasets [12–14]. Following these factor analyses, Hierarchical Clustering on Principal Components (HCPC) was applied within each block to identify clusters of veterinarians sharing similar characteristics. For each HCPC, the number of dimensions retained for clustering was determined by the criterion of explaining at least 80% of the cumulative block inertia [15]. The optimal number of clusters was then determined using a combination of criteria, including visual inspection of dendrograms, inertia gain plots (elbow method), and interpretability of the resulting clusters in terms of veterinary profiles and practices.

To explore broader patterns across all dimensions of the study and to develop an overall profile associated with PRRS control, a final MCA was performed using the categorical clusters derived from each block. In this global analysis, the variable “country” was included as a supplementary variable in order to visualize its association with the identified clusters, without influencing the clustering process itself. This allowed us to account for potential contextual effects related to geographical differences, while avoiding any bias due to unequal sample sizes across countries.

### Software

This survey was developed using Sphinx iQ3 (v8.2.2). Collected answers were managed in Microsoft Excel v14.0.6 (2010) and analysed with R Studio (v2023.06.05). Each block was analysed separately using the “FactoMineR” and “factoextra” packages in R (Husson et al. 2009). Specifically, PCA was performed using the PCA() function for continuous variables, MCA was performed using the MCA() function for categorical variables and, FAMD suitable for datasets combining quantitative and qualitative variables, was conducted using the FAMD() function. For unsupervised clustering, HCPC was applied using the HCPC() function, following dimension reduction by PCA, MCA or FAMD. Visualization and interpretation of the results were supported by functions from the “factoextra” package, such as fviz_pca_ind(), fviz_pca_var(), fviz_mca_biplot(), and others.

## Results

### General results

A total of 108 swine veterinarians from 22 European countries completed the survey, representing an estimated response rate of 18% based on the initial distribution list. Most respondents were practicing veterinarians with direct on-farm responsibilities, although a small number also reported part in diagnostics, consultancy, or administrative tasks. Veterinarians were distributed across a range of employment types (private practitioners, salaried veterinarians), years of experience, and number of employees in the practice. The highest number of responses came from France (*n* = 34), Denmark (*n* = 10), The Netherlands (*n* = 9).

### Veterinary profiles

#### Sociodemographic veterinary profile

Three major profiles (= clusters) of swine veterinarians based on sociodemographic features were identified (Appendix 2). Each cluster is described in Table 1. The “Early career vets” (*N* = 18) comprised veterinarians under 40 years-old, with a mean age of 30.8 years. All had less than 10 years of experience and were more likely to be salaried employees in small to medium-sized practices (less than 50 employees). The second group (*N* = 42), the “Large practice vets” worked in companies with over 50 employees. This group had a higher proportion of women and more national specialization credentials than the previous group. Despite being older on average (mean age: 47 years), some members had relatively few years of experience due to career transitions. Then, the “Experienced private practitioners” included self-employed veterinarians with over 10 years of experience, working in small practices with less than 10 employees. They had the highest rate of national specialization (52%) and were almost exclusively male (Table 1).

**Table 1:** Characteristics of the three sociodemographic profiles

Sociodemographic variables	Early career vets (SC1)	Large practice vets (SC2)	Experienced private practitioners (SC3)	Overall
Number per group	*N* = 18	*N* = 42	*N* = 48	*N* = 108
Sex ratio (%male-%female)	72% − 28%	38% − 62%	71% − 29%	58%-42%
Age (mean ± SD)	30.83 ± 5.61	46.98 ± 8.58	49.50 ± 10.04	45.40 ± 11.03
Proportion of vets with less than 10 years of experience	100%	11.90%	0.00%	21.30%
Proportion of vets working as salaried	66.67%	83.33%	22.92%	53.70%
Company size:				
Less than 10 employees	44.44%	7.14%	47.92%	31.48%
Between 10–50 employees	50.00%	26.19%	45.83%	38.89%
More than 50 employees	5.56%	66.67%	6.25%	29.63%
Other diploma (European specialist/National specialisation)	33.33% (11% / 22%)	52.38% (9% / 43%)	62.50% (10% / 52%)	58.33% (10% / 44%)

SD: Standard deviation

#### Exchanges and professional interactions profiles

Three distinct patterns of professional interactions were found (Table 2). First, the “Independent learners” prioritized personal education, spending significant time in conferences, training, and reading scientific literature. They had limited interaction with laboratories (both analysis and diagnosis ones) and rarely participated in collaborative performance reviews and/or structured meetings. The “Collaborative practitioners” frequently exchanged information with peers and/or authorities. Although not frequently interacted with laboratories, they emphasized communication and feedback within the professional network. Finally, the “Networked experts” was composed of veterinarians who maintained strong connections with laboratories (both analysis and diagnosis), regularly attended team meetings, and made extensive use of performance indicators. Teamwork and data analysis were central to their practice profile. The ways in which veterinarians interact with peers, laboratories, and institutional actors appear to shape their learning strategies and openness to innovation. The division of veterinarians based on interaction styles suggests different paths for continuing education and knowledge dissemination (Appendix 3).

**Table 2:** Characteristics of the three profiles regarding veterinarians’ exchanges and professional interactions

Exchanges and professional interactions variables	Independent learners (EXC3)	Collaborative practitioners (EXC1)	Networked experts (EXC2)	Overall
Number per group	*N* = 17	*N* = 46	*N* = 45	*N* = 108
Within your company, how often are meetings held to develop common procedures (guidelines)? (0 = Never, 4 = Very often)	2.59 ± 1.28	2.15 ± 1.63	1.84 ± 1.61	2.09 ± 1.58
Do you usually work as part of a team within your organization? If yes, at which step(s) do you consider teamwork necessary? (Assign a score from 1 to 5 to each item below) ^1^ *Implementation of diagnostic procedures* *Analysis of technical and economic data* *Farm monitoring support* *Interpretation of laboratory test results*	1.49 ± 0.89	1.53 ± 1.13	1.89 ± 1.00	1.68 ± 1.05
How frequently do you interact with the following peers? (Scale: 0 = Rarely or never, 1 = Occasionally, 2 = Often, 3 = Very often)				
Veterinarians and technicians from pharmaceutical companies and from diagnostic laboratories^2^ Other veterinarians Other technicians Public administration representatives	0.43 ± 0.46 0.88 ± 0.70 1.06 ± 1.20 0.00 ± 0.00	0.55 ± 0.30 2.20 ± 0.98 0.09 ± 0.28 0.48 ± 1.03	1.23 ± 0.23 0.73 ± 0.65 0.07 ± 0.25 0.04 ± 0.30	0.83 ± 0.46 1.38 ± 1.07 0.23 ± 0.64 0.22 ± 0.73
How frequently do you engage in the following activities? (Please express your answer as a percentage of your working time: *reading new publications*,* attending congresses/seminars*,* participating in training sessions*)^3^	35.75%	7.90%	8.27%	12.44%
In the farms you regularly oversee, do you usually monitor technical and economic performance indicators on a regular basis? (Scale: 0 = No, 1 = It depends, 2 = Yes)	0.94 ± 0.97	1.89 ± 0.38	1.69 ± 0.61	1.66 ± 0.64

^1^Mean score (from 1 to 5) reflects how often teamwork is considered necessary across the different steps

^2^For these two categories (veterinarians and technicians from pharmaceutical companies, and from diagnostic laboratories), individual scores were averaged to create a single composite score reflecting overall frequency of interaction with both professional types

^3^A mean score was calculated across the three items to reflect the overall time dedicated to continuous professional development

#### EBVM practices profiles

Three attitudinal clusters toward EBVM were identified (Appendix 4). Our results showed veterinarians who are “open” to EBVM, they accepted EBVM principles and believed they have already integrated it into daily practice. They reported minimal resistance and frequently referred to scientific literature. The “sceptical” practitioners acknowledged the value of EBVM, they perceived that it was not sufficiently implemented in the field. Structural barriers and lack of resources were cited as key obstacles. Finally, “reluctant” practitioners group expressed doubts about the practicality and relevance of EBVM in field conditions. Their resistance reduced from perceived incompatibility between theory and real-world constraints, as well as scepticism about the evidence base itself. The distributions of EBVM attitudes are described in Table 3.

**Table 3:** Characteristics of the three profiles regarding EBVM knowledge and perceptions

EBVM knowledge and perceptions variables	Open (EBM1)	Sceptical (EBM2)	Reluctant (EBM3)	Overall
Number per group	*N* = 70	*N* = 19	*N* = 19	*N* = 108
Percentage of respondents familiar with the concept of “Evidence-Based Veterinary Medicine” (number)	68.57% (48)	73.68% (14)	57.89% (11)	67.59% (73)
Percentage of veterinarians who perceived usefulness of the EBM approach for clinical decision-making (number)	100% (70)	100% (19)	0% (0)	82.41% (89)
Percentage of veterinarians who expressed reservations or obstacles (number)	0% (0)	100% (19)	26.32% (5)	22.22% (24)
Percentage of respondents who believed EBVM is sufficiently developed in daily practice (number)	51.43% (36)	52.63% (10)	26.32% (5)	47.22% (51)

#### Veterinary profiles regarding PRRS context and practices

Two clusters emerged from the MCA focused on PRRS context, available tools and management practices (Appendix 5). The first, “Technically engaged”, includes veterinarians who actively engage in PRRS control, have broad access to MLV and inactivated vaccines, and face ongoing but manageable challenges such as diagnostic limitations. The second, referred to as “Low intervention”, includes veterinarians with limited or no engagement in stabilization efforts, often associated with the absence of PRRS cases, reduced diagnostic practices, and poor access to vaccines. These contrasting profiles reflect differences in both the relevance of PRRS management and the capacity or willingness to implement PRRS control strategies (Table 4).

**Table 4:** Characteristics of the three profiles regarding PRRS context and practices

PRRS context and practices variables	Technically engaged (PRRS+)	Low intervention (PRRS-)	Overall
Number per group	*N* = 93	*N* = 15	*N* = 108
Observed prevalence of PRRS (in %)	61.30%	15.33%	54.92%
Genotype of PRRSV present in the practice area of the respondent:			
Type I	76.34% (71)	0.00%	65.74% (71)
Type II	2.15% (2)	0.00%	2.15% (2)
Bot	17.20% (16)	20.00% (3)	17.60% (19)
None	4.30% (4)	80.00% (12)	14.81% (16)
Access to diagnostic and analysis laboratory	95.70% (89)	93.33% (14)	95.37% (103)
Access to partial sequencing (ORF7 and/or ORF5)	91.40% (85)	33.33% (5)	83.33% (90)
Access to full genome sequencing	61.29% (57)	33.33% (5)	57.41% (62)
Proportion of respondents frequently encountering PRRS cases	32.26% (30)	6.67% (1)	28.70% (31)
Availability of sufficient information and tools for diagnostic decision-making in:			
Breeding sector	97.85% (91)	66.67% (10)	93.51% (101)
Post-weaning sector	94.62%(88)	73.33% (11)	91.67% (99)
Access to MLV1^a^	89.25% (83)	20.00% (3)	79.63% (86)
Access to MLV2^b^	25.81% (24)	0.00% (0)	25.81% (24)
Access to inactivated vaccines	63.44% (59)	0.00% (0)	63.44% (59)
Access to autogenous vaccines	6.45% (6)	0.00% (0)	6.45% (6)

^a^ PRRSV-1 Modified Live vaccine

^b^ PRRSV-2 Modified Live vaccine

#### Veterinary profiles regarding PRRSV stabilization programs

Veterinarians were grouped into four clusters based on their responses about PRRSV stabilization experience (Appendix 6 and Table 5). The first one was the ‘successful group’, i.e. veterinarians who had successfully implemented stabilization protocols (100% of success), often reporting access to adequate tools, cooperative farmers, and institutional support. They exemplified a structured and proactive approach to PRRS management. Two ‘partial success groups’ were identified according to the main reason being either:

(i) Farmer limitations, these veterinarians having attempted stabilization but faced challenges due to inconsistent farmer cooperation. Despite having technical knowledge, implementation was hindered by sociocultural factors on farms;
(ii) Technical limitations, this group had support from farmers but lacked the diagnostic tools or institutional backing necessary to implement effective protocols.

Finally, the last one was composed of veterinarians who had not initiated any stabilization programme. Reasons varied from lack of opportunity, perceived inefficacy, or contextual constraints. The typology reveals that PRRS control success is a multidimensional outcome, contingent upon the convergence of technical capacity (e.g., diagnostics, vaccine access) and relational alignment (e.g., farmer compliance, professional trust). The absence of either component appears to be a limiting factor across contexts.

**Table 5:** Characteristics of the three profiles regarding PRRSV stabilization programmes experiences

PRRSV stabilization programmes experiences variables	Total success (Stab1)	Partial success due to farmer limitations (Stab2)	Partial success due to technical limitations (Stab3)	Never implemented (Stab4)
Number per group	*N* = 17	*N* = 36	*N* = 19	*N* = 36
Success rate of implemented PRRSV stabilization programs	99.41%	64.00%	49.89%	NA
Lack to achieve 100% success:				
Insufficient tools	0% (0)	0% (0)	42.11% (8)	NA
Insufficient scientific evidence	0% (0)	0% (0)	57.89% (11)	NA
Limited engagement from the farmer	0% (0)	100% (36)	47.37% (9)	NA

NA: Not applicable

### Cross-profiles in PRRSV stabilization programmes’ success

**Fig. 2:**
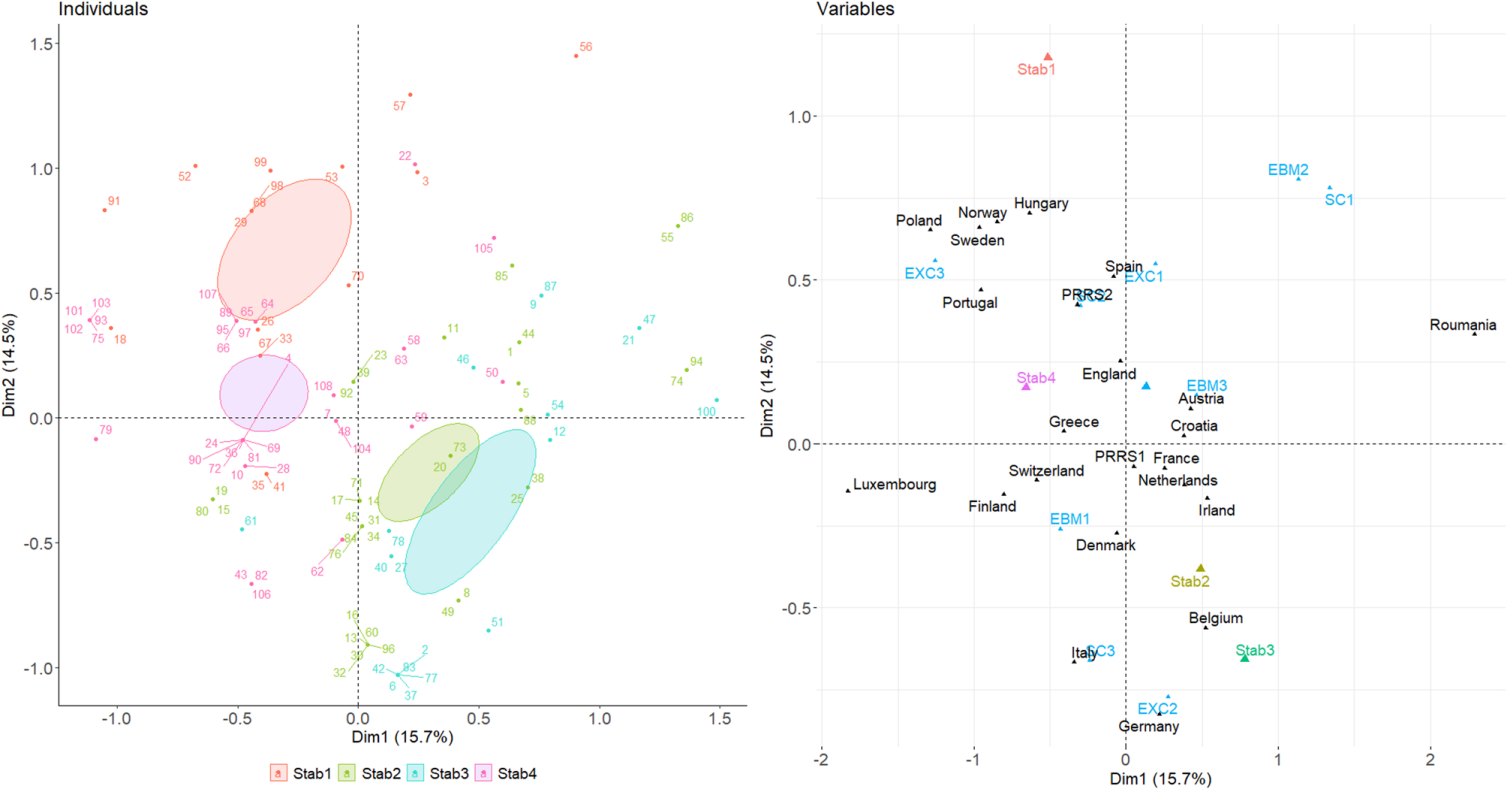
Illustration of the four final profiles of the swine veterinarians. MCA biplot illustrating the distribution of individuals and the contribution of categorical and continuous variables. The individuals are represented as points, while categorical variables are displayed as vectors. The plot shows the relationships between the first and second principal components, with continuous variables shown on the axes and categorical variables color-coded for clarity. The variance explained by each component is also indicated in the inset. The concentric ellipses are defined by a 95% confidence interval (around group mean points or barycentre). The figure highlights the positioning of predefined clusters resulting from the multidimensional analysis, grouped according to each thematic block: SC1 (Early career vets), SC2 (Large practice vets), and SC3 (Experienced private practitioners) for sociodemographic characteristics; EXC1 (Collaborative practitioners), EXC2 (Networked experts), and EXC3 (Independent learners) for exchanges and professional interactions; EBM1 (Open), EBM2 (Sceptical), and EBM3 (Reluctant) for EBVM knowledge and perceptions; and PRRS+ (Technically engaged) and PRRS- (Low intervention) for PRRS context and practices

The objective was to explore overarching associations between the veterinarian profiles previously identified across thematic blocks. This analysis highlighted several proximities between these profiles, as illustrated in Fig. 2. Seven dimensions were extracted, explaining a cumulative variance of 85.61%. The first three dimensions, which together accounted for 42.6% of the total variance, were retained for interpretation based on both their explanatory contribution and the clarity of the patterns they revealed.

Dimension 1 (15.7% of variance) was primarily shaped by variables related to PRRSV stabilization strategies (particularly categories Stab2, Stab3, and Stab4) and the EBVM mindset, especially EBM1 (“Open” group) and EBM2 (“Sceptical” ones). Categories representing full implementation (Stab1_Total success) and partial implementation due to technical limitations (Stab3_Technical limitations) were strongly opposed along this axis, suggesting that the first dimension primarily differentiates veterinarians according to their capacity to achieve successful stabilization outcomes. This contrast appears to reflect deeper structural inequalities: veterinarians in the “total success” group are often embedded in well-resourced environments, with reliable access to diagnostic tools and a collaborative professional ecosystem, whereas those in the “technical limitations” group tend to operate in settings with constrained infrastructures, despite high individual motivation or farmer cooperation. The axis thus captures not only outcome differences, but also the underlying systemic enablers and barriers to effective PRRS control. Notably, SC1 (“Early career vets”) and EXC3 (“Independent learners”) also showed high contributions, suggesting that lack of peer interaction and a specific professional profile are associated with low or failed implementation’s success.

Dimension 2 (14.5%) captured contrasts between collaborative engagement and institutional affiliations, particularly through the high contributions EXC1, EXC2, and SC3. This dimension is mostly associated with Stab1 (Total success of PRRSV stabilization programmes). Practitioners with low lab interaction but frequent peer exchanges (e.g., EXC1) were distinct from those with limited collaboration or more isolated working patterns. The influence of national context was also visible, as supplementary categories like Belgium, France, and Spain showed significant projections on this axis, indicating that country-level differences in professional ecosystems may also shape attitudes and behaviours.

Dimension 3 (12.4%), although marginally less explanatory than the first two, provides additional insights into how stabilization practices intersect with sociodemographic and attitudinal patterns. This axis notably separates veterinarians who have successfully implemented PRRSV stabilization programmes (Stab1) from those who have never attempted such interventions (Stab4), with the former positioned negatively and the latter positively along the dimension. This contrast appears to align with generational and attitudinal divides: Stab1 is associated with EBVM-enthusiastic and well-connected veterinarians, while Stab4 aligns with more isolated or less engaged profiles, including a higher representation of EBM3 (“reluctant”) and EXC3 (“independent learners”). While technical capacity remains essential, the influence of mindset and structural integration appears to persist as a determinant of whether stabilization programmes are initiated at all.

Indeed, several final profiles across stabilization outcomes further supports the results obtained from the three dimensions analysis (Table 6). Veterinarians achieving total success (Stab1) are predominantly experienced practitioners embedded in large practices (SC2 and SC3), actively engaged in collaborative networks (especially EXC1 and EXC2), and strongly aligned with an evidence-based mindset (notably EBM1 “Open”). In contrast, those who did not implement stabilization programmes (Stab4) are more frequently represented among early-career vets (SC1), independent learners (EXC3), and those expressing reluctance toward EBVM (EBM3 “Reluctant”). Partial success scenarios (Stab2 and Stab3) reveal more diverse configurations, often shaped by external constraints such as limited farmer cooperation or technical limitations. These patterns suggest that successful PRRSV stabilization is not solely driven by technical expertise, but also by broader structural enablers, peer interaction, and a positive orientation toward evidence-based practice.

**Table 6:** Characteristics of the final profiles

	Total success (Stab1)	Partial success due to farmer limitations (Stab2)	Partial success due to technical limitations (Stab3)	Not implemented (Stab4)
**Sociodemographic characteristics**				
SC1 – Early career vets	17.65%	22.22%	15.79%	11.11%
SC2 – Large practice vets	47.06%	30.56%	31.58%	47.22%
SC3 – Experienced private practitioners	35.29%	47.22%	52.63%	41.67%
**Exchanges and professional interactions**				
EXC1 – Collaborative practitioners	58.82%	41.67%	36.84%	38.89%
EXC2 – Networked experts	17.65%	50.00%	57.89%	36.11%
EXC3 – Independent learners	23.53%	8.33%	5.26%	25.00%
**EBVM knowledge and perceptions**				
EBM1 – Open	70.59%	66.67%	52.63%	66.67%
EBM2 – Sceptical	11.76%	19.44%	26.32%	13.89%
EBM3 – Reluctant	17.65%	13.89%	21.05%	19.44%
**PRRS context and practices**				
PRRS+ – Technically engaged	100.00%	100.00%	89.47%	63.89%
PRRS- – Low intervention	0.00%	0.00%	10.53%	36.11%

Percentages indicate the distribution of veterinarian sub-profiles across each PRRSV stabilization outcome

## Discussion

The results of this study highlight a diversity of profiles among veterinary practitioners in Europe, particularly regarding their involvement in EBVM, their professional interactions, and their practices in managing PRRS. This heterogeneity suggests that PRRS management is influenced by a combination of individual, structural, and contextual factors that vary considerably between veterinarians. This diversity is pivotal to understand the challenges of PRRS control in practice.

While this study provides valuable insights into the practices and attitudes of European veterinarians, several limitations must be considered. As previously documented in veterinary survey researches, proactive or academically oriented veterinarians are more likely to respond, leading to possible overrepresentation of EBVM-oriented profiles [16, 17]. The moderate response rate (18%) and the potential selection bias towards veterinarians strongly involved in PRRS management may limit the generalizability of the results to the entire profession. Indeed, veterinarians who chose to participate in the survey may be those with a particular interest in PRRS or in evidence-based approaches, which could skew the findings toward more engaged, better-resourced, or more proactive professionals. However, it is worth noting that 36 of the 108 veterinarians who completed the survey had never implemented PRRSV stabilization programmes (Stab4). This suggests that the sample is not exclusively composed of highly engaged or proactive professionals but also includes individuals potentially less concerned or less experienced with the topic, thereby mitigating the risk of strong selection bias. Their inclusion provides important contrastive insights into the barriers to implementation and the diversity of professional profiles within the field. Thus, while the moderate overall response rate and the potential overrepresentation of PRRS-engaged practitioners remain valid concerns, the presence of these less-involved respondents enhances the credibility and balance of the findings, capturing a wider range of perspectives and levels of engagement.

As a result, certain profiles, such as those who are less familiar with PRRS control strategies, more sceptical of EBVM, or working in more isolated or resource-constrained settings, may be underrepresented in the sample. This potential overrepresentation of highly engaged practitioners could lead to an overestimation of the adoption and integration of PRRS control strategies within the broader veterinary population. Additionally, the study relies on self-reported evaluations from veterinarians, which may introduce response bias. However, this is the first study involving a wide range of European swine veterinarians and deciphering among sociodemographic characteristics, EBVM knowledge and application, PRRS context and management practices, which factors influenced their experience with PRRSV stabilization programmes. Moreover, it is also important to note that veterinarians from PRRSV-free countries were included in the study population. Their responses contributed to the identification of the “low intervention” profile, which offers a meaningful contrast to veterinarians working in endemic areas. Including these respondents was relevant because even in countries officially free from PRRSV, veterinarians remain engaged in preventive strategies, surveillance, and biosecurity efforts aimed at maintaining freedom from the disease. It is also to be noticed, these veterinarians who have their main area of activities in a PRRSV-free country can consult pig farms in other areas that are not free from PRRSV. Their perspectives therefore provide valuable insights into structural and contextual differences in veterinary practice that are not solely determined by the presence of PRRSV.

The analysis of sociodemographic profiles revealed three main groups of veterinarians: the early-career veterinarians, the veterinarians in large practices and the experienced veterinarians in private practice. Younger veterinarians, typically in salaried positions and with fewer specialization credentials, may face constraints related to decision-making autonomy, diagnostic tool access, or targeted training in complex disease management [18, 19]. These limitations can significantly impact their ability to manage clinical cases all alone [20]. In contrast, veterinarians in larger practices or those with more experience seem to benefit from a stronger support network and greater professional autonomy, enabling them to take a more proactive role in disease management and in integrating PRRS control strategies. Similar segmentation approaches have been adopted in other livestock sectors. For instance, Kristensen and Enevoldsen (2008) identified differentiated veterinary profiles based on values and communication styles in dairy herd management strategies, while Crawford et al. (2024) documented the emergence of distinct expert and advisory roles among ovine practitioners in the UK [21, 22]. These findings echo our own typology, reinforcing the idea that professional identity, relational patterns, and context-specific constraints jointly shape veterinarians’ approach to disease control, regardless of species. However, as noted in previous research, even experienced veterinarians face challenges related to the availability of diagnostic tools or managing the cooperation of farmers [23]. Specifically, access to effective vaccines and diagnostic tools is crucial for effective PRRS management, and the disparity between veterinarians in their access to these tools could explain some inconsistencies in the application of control strategies. Indeed, recent qualitative research has shown that veterinarians may hold ambivalent views toward the integration of data-driven technologies, often citing concerns about usability, data overload, and relevance to on-farm realities, which may further complicate the practical application of stabilization programmes [24]. Moreover, similar patterns of opinion segmentation have been observed beyond veterinarians, as farmers also display diverse perspectives towards disease eradication programmes, such as in the Spanish Tuberculosis Eradication Programme where both farmers and veterinarians showed varied attitudes that influenced the programme’s implementation and success [25].

The three identified profiles of professional interaction offer interesting insight into how veterinarians’ access and utilize scientific knowledge. “Independent learners” who prioritize self-directed learning and individual consumption of knowledge, may struggle more to apply EBVM principles due to a lack of institutional support and limited communication with other veterinarians or laboratories. This is consistent with findings from Leasure et al. (2008), who emphasize that environments fostering interprofessional collaboration significantly improve EBVM adoption [26]. However, “collaborative practitioners” and “networked experts” appear to income from a stronger network, facilitating the integration of scientific knowledge into their daily practices, particularly through regular exchanges with peers, authorities, laboratories and multidisciplinary teams. Collaboration with other veterinarians and teamwork, as highlighted in the analysis, seems especially important in managing complex diseases like PRRS, which control requires a collaborative approach and regular sharing of information. Moreover, the diversity of professional interaction profiles raises important questions about how education and knowledge dissemination strategies could be adapted to improve EBVM application in veterinary practice.

Indeed, the groups of attitudes toward EBVM illustrate significant differences in how veterinarians perceive EBVM and its application in PRRS management [5]. The division between “open” and “reluctant” veterinarians is particularly important for PRRS management, as consistent and informed implementation of control strategies, such as stabilization programmes, requires strong engagement from veterinarians. Practitioners expressing scepticism towards EBVM often cite poor field applicability or lack of context-relevant data as reasons for non-adoption [27].

Then, two profiles of PRRS management reveal marked differences in the application of control strategies. Technically engaged veterinarians have access to advanced diagnostic tools and vaccines and are more likely to implement stabilization programmes to control PRRSV. This group seems better equipped to address the technical challenges posed by PRRSV, but it is important to note that even within this group, access to resources and farmer cooperation are crucial factors for the success of stabilization programmes. These discrepancies may reflect national inequalities in veterinary infrastructure, which have been shown to directly influence PRRS control success rates [28]. Conversely, “low intervention” veterinarians, often facing difficulties accessing diagnostic tools and vaccines, are less well equipped to implement stabilization strategies, despite acknowledging the need to control PRRS. This group reflects a shared reality among many veterinarians in Europe, where inequalities in access to resources and a lack of institutional support may limit the effectiveness of disease management efforts. Furthermore, it is important to note that the success rates of PRRSV herd stabilization reported in this study were based on the own evaluation by each veterinarian. No standardized definition of “success” was provided to respondents, as the aim was to capture their personal perception of program outcomes in real-world conditions. This approach inevitably introduces variability, as different practitioners may interpret success differently depending on their own experience, herd context, and practical constraints. Future studies could complement these findings by integrating objective virological or epidemiological data to validate or calibrate perceived success.

## Conclusion

This study shows an important diversity of swine veterinarians’ profiles regarding several thematic such as sociodemographic characteristics, professional exchanges, EBVM perceptions, PRRS context, etc. These results help us to understand how management of PRRS through stabilization programmes can be influenced by several externalities linked to veterinarians’ profile. This heterogeneity underscores the importance of better considerate veterinarians’ profiles and practices to develop more tailored knowledge dissemination and training strategies that address the various barriers to PRRSV stabilization programmes’ implementations. A more targeted approach, based on the specific needs of each veterinarian group, could improve the effectiveness of control strategies and contribute to a more informed and coherent approach to PRRS management. In complement to this survey, it might be beneficial to set up more in-depth field studies involving direct observation of practices and interviews with industry stakeholders. Indeed, future studies could consider the economic aspects of PRRS control, such as weighing the costs of control programs against the potential losses from disease in a stabilized herd, to better understand veterinarians’ approach.

## Supplementary Information

Below is the link to the electronic supplementary material.

Supplementary Material 1

## Data Availability

The datasets used and/or analysed during the current study are available from the corresponding author on reasonable request.
