# Supplementary material for: PRRS management by European swine veterinarians: a descriptive insight into practices and profiles
Source: Porcine Health Manag. 2025 Nov 5;11:55. doi: 10.1186/s40813-025-00467-0 (PMC12587695; doi:10.1186/s40813-025-00467-0)
Supplement: Supplementary file 1 — Supplementary Material 1 [file 40813_2025_467_MOESM1_ESM.docx]

**Appendix 1. Variable dictionary**

|  | **Variable name** | **Description** | **Variable type** | **Values / Units** |
| --- | --- | --- | --- | --- |
|  | **Client ID** | Unique identifier for each respondent | Categorical (nominal) | Alphanumeric code |
| **Sociodemographic characteristics** | **Gender** | Self-identified gender | Categorical (nominal) | Male / Female / Other |
|  | **Age** | Respondent’s age | Quantitative (continuous) | Years |
|  | **Other diploma** | Additional degrees or certifications obtained | Categorical (nominal) | National specialization / European certificate / Other (multiple responses possible) |
|  | **Status** | Current employment type | Categorical (nominal) | Private practitioner / Employee |
|  | **Company size** | Size of the employing organization | Ordinal | <5 employees / 5–10 employees / 10–50 employees / >50 employees |
|  | **Vets’ proportions** | Proportion of veterinarians in the organization | Quantitative (continuous) | Percentage (0 to 100%) |
|  | **Years of exp.** | Years of experience working in swine production | Categorical (nominal) | <5 years / 5–10 years / >10 years |
|  | **Contract** | Employment status | Categorical (nominal) | Full-time / Part-time |
|  | **Swine proportion** | Share of work activities dedicated to pig consulting | Quantitative (continuous) | Percentage (0 to 100%) |
|  | **Country** | Country in which the respondent mostly works | Categorical (nominal) | One of 30 listed European countries (e.g., France, Germany, Spain, etc.) |
| **Professional interactions & exchanges** | **Meeting frequencies** | Do you usually work in a team within your organization? | Categorical (ordinal) | Never (0) – Very often (4) |
|  | **Teamwork** | Teamwork for Implementation of diagnosis / Techno-economic analysis / Herd monitoring / Lab result interpretation | Categorical (ordinal, ranked) | From 1 to 4 (ordered) |
|  | **Laboratories exchanges** | Frequency of interaction with each peer type | Categorical (ordinal) | Rarely (1) – Very often (4) |
|  | **Vets’ exchanges** | Frequency of interaction with each peer type | Categorical (ordinal) | Rarely (1) – Very often (4) |
|  | **Technicians’ exchanges** | Frequency of interaction with each peer type | Categorical (ordinal) | Rarely (1) – Very often (4) |
|  | **Authorities’ exchanges** | Frequency of interaction with each peer type | Categorical (ordinal) | Rarely (1) – Very often (4) |
|  | **Continuing education** | Frequency of involvement in evidence-based activities (reading publications, attending congresses/seminars, trainings) | Numerical (percentage) | 0 to 100% of working time |
|  | **Performance monitoring** | Technical and economic performance indicators monitoring | Categorical (nominal) | Yes / No / It depends |
| **Evidence-Based Veterinary Medicine** | **Knowledge** | Had you heard of Evidence-Based Medicine (EBM) before we contacted you? | Categorical (binary) | Yes / No |
|  | **Usefulness** | Do you find this approach useful in supporting decision-making? | Categorical (nominal) | Yes / No / It depends |
|  | **Barriers** | Do you have any reluctance or barriers to using the EBM approach? | Categorical (binary) | Yes / No |
|  | **Development in practice** | In your opinion, is the EBM approach sufficiently developed in daily practice? | Ordinal (Likert) | 1 = Strongly disagree /  2 = Somewhat disagree /  3 = Somewhat agree / 4 = Strongly agree |
| **PRRSV management and practices** | **Prevalence** | Estimated prevalence of PRRSV in the respondent’s country | Quantitative (continuous) | Percentage (0 to 100%) |
|  | **Genotype** | PRRSV genotypes encountered | Categorical (nominal) | Type I / Type II / Both / None |
|  | **Sampling** | Who primarily performs the biological samplings | Categorical (nominal) | Farmer / Myself / Technician / Other |
|  | **Lab access** | Easy access to diagnostic laboratory facilities | Categorical (binary) | Yes / No |
|  | **Partial sequencing** | Access to partial sequencing (e.g., ORF5 and/or ORF7) | Categorical (nominal) | Yes / No / Don’t know |
|  | **Whole genome sequencing** | Access to full genome sequencing | Categorical (nominal) | Yes / No / Don’t know |
|  | **PRRSV cases** | Frequency of PRRSV clinical cases encountered in practice | Ordinal (Likert) | 0 = Never /  1 = Rarely /  2 = Occasionally  (<6 cases/year) /  3 = Often  (≥1 case/month) /  4 = Very often |
|  | **Diagnostic procedure** | Presence of predefined diagnostic procedures | Categorical (binary) | Yes / No |
|  | **Company consensus for diagnostic** | Basis of diagnostic procedures | Categorical (binary) | Yes / No |
|  | **Regulatory recommendations for diagnostic** | Basis of diagnostic procedures | Categorical (binary) | Yes / No |
|  | **Professional consensus for diagnostic** | Basis of diagnostic procedures | Categorical (binary) | Yes / No |
|  | **Sow diagnostic tools** | Sufficiency of resources to diagnose in sows | Categorical (binary) | Yes / No |
|  | **Piglets’ diagnostic tools** | Sufficiency of resources to diagnose in post-weaning/fattening pigs | Categorical (binary) | Yes / No |
|  | **MLV1 access** | Access to PRRS vaccines | Categorical (binary) | Yes / No |
|  | **MLV2 access** | Access to PRRS vaccines | Categorical (binary) | Yes / No |
|  | **Inactivated vaccine access** | Access to PRRS vaccines | Categorical (binary) | Yes / No |
|  | **Autogenous vaccine access** | Access to PRRS vaccines | Categorical (binary) | Yes / No |
|  | **Vaccine regulations** | Whether vaccination is subject to regulation | Categorical (binary) | Yes / No |
|  | **Live vaccine for sows?** | Recommendation to use inactivated vaccine alone in sows | Categorical (nominal) | Yes / No / No opinion |
|  | **MLV for sows?** | Recommendation to use live vaccine alone in sows | Categorical (nominal) | Yes / No / No opinion |
|  | **Both combine for sows?** | Recommendation to use both types in combination | Categorical (nominal) | Yes / No / No opinion |
|  | **Effective live vaccine for sows?** | Perceived efficacy of inactivated vaccine alone in sows | Categorical (ordinal) | 1 = Not effective at all to 4 = Very effective |
|  | **Effective MLV for sows?** | Perceived efficacy of live vaccine alone in sows | Categorical (ordinal) | 1 = Not effective at all to 4 = Very effective |
|  | **Effective both for sows?** | Perceived efficacy of combined vaccination in sows | Categorical (ordinal) | 1 = Not effective at all to 4 = Very effective |
|  | **Live vaccine for piglets?** | Recommendation to use inactivated vaccine alone in post-weaning/fattening pigs | Categorical (nominal) | Yes / No / No opinion |
|  | **MLV for piglets?** | Recommendation to use live vaccine alone in post-weaning/fattening pigs | Categorical (nominal) | Yes / No / No opinion |
|  | **Both combine for piglets?** | Recommendation to use both types in combination | Categorical (nominal) | Yes / No / No opinion |
|  | **Effective live vaccine for piglets?** | Perceived efficacy of inactivated vaccine alone in post-weaning/fattening pigs | Categorical (ordinal) | 1 = Not effective at all to 4 = Very effective |
|  | **Effective MLV for piglets?** | Perceived efficacy of live vaccine alone in post-weaning/fattening pigs | Categorical (ordinal) | 1 = Not effective at all to 4 = Very effective |
|  | **Effective both for piglets?** | Perceived efficacy of combined vaccination in post-weaning/fattening pigs | Categorical (ordinal) | 1 = Not effective at all to 4 = Very effective |
|  | **Vaccine combination** | Combined use of multiple live modified vaccines in a single farm | Categorical (binary) | Yes / No |
| **PRRSV stabilization programs** | **Stabilization program** | Have you ever implemented stabilization programs? | Categorical (binary) | Yes / No |
|  | **%succes** | Estimated success rate of stabilization programs in the last two years (%) | Quantitative (continuous) | 0 to 100% |
|  | **Lack of tools** | Technical tools (apps, calculators, etc.) were missing to obtain 100% of success | Categorical (binary) | Yes / No |
|  | **Lack of farmer cooperation** | Farmer cooperation was missing to obtain 100% of success | Categorical (binary) | Yes / No |
|  | **Lack of evidence** | Scientific arguments or evidence were missing to obtain 100% of success | Categorical (binary) | Yes / No |

**Appendix 2. Illustration of the three sociodemographic profiles of the swine veterinarians**


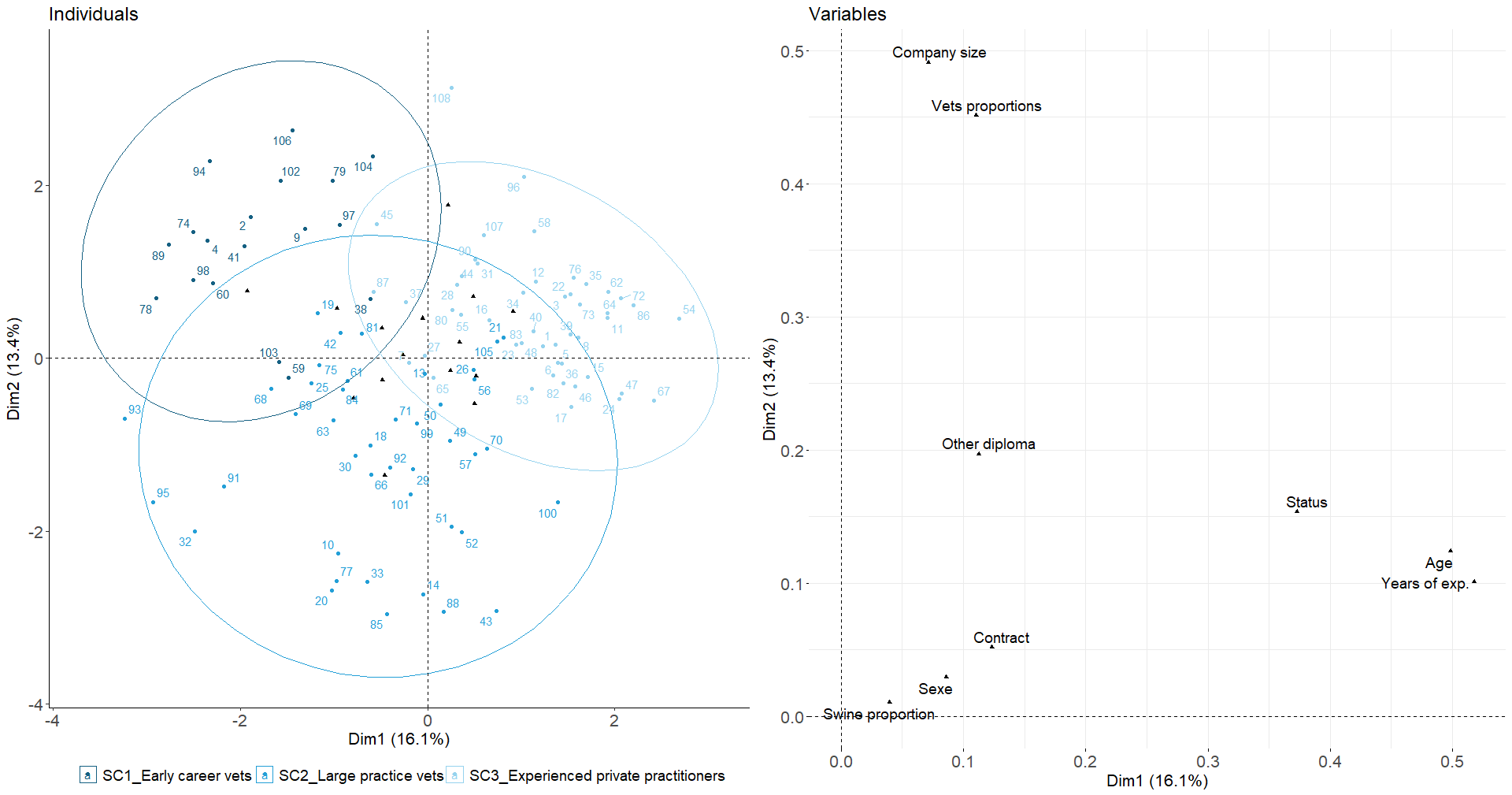


*Legend: FAMD biplot illustrating the distribution of individuals and the contribution of categorical and continuous variables. The individuals are represented as points, while categorical variables are displayed as vectors. The plot shows the relationships between the first and second principal components, with continuous variables shown on the axes and categorical variables color-coded for clarity. The variance explained by each component is also indicated in the inset.*

**Appendix 3. Illustration of the three profiles of the swine veterinarians regarding their professional exchange and interaction**


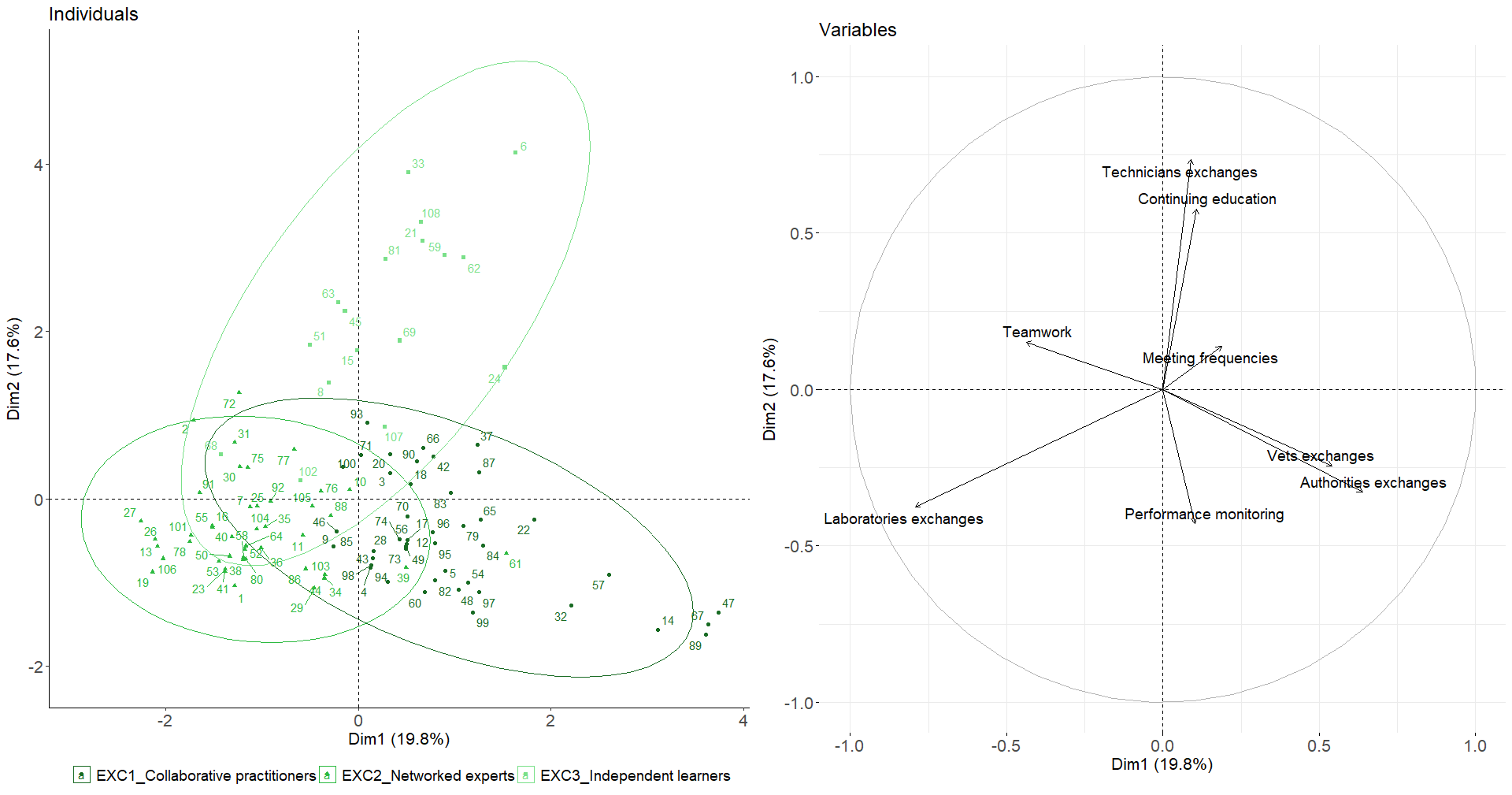


*Legend: PCA biplot illustrating the distribution of individuals and the contribution of categorical and continuous variables. The individuals are represented as points, while categorical variables are displayed as vectors. The plot shows the relationships between the first and second principal components, with continuous variables shown on the axes and categorical variables color-coded for clarity. The variance explained by each component is also indicated in the inset.*

**Appendix 4. Illustration of the three profiles of the swine veterinarians regarding EBVM**


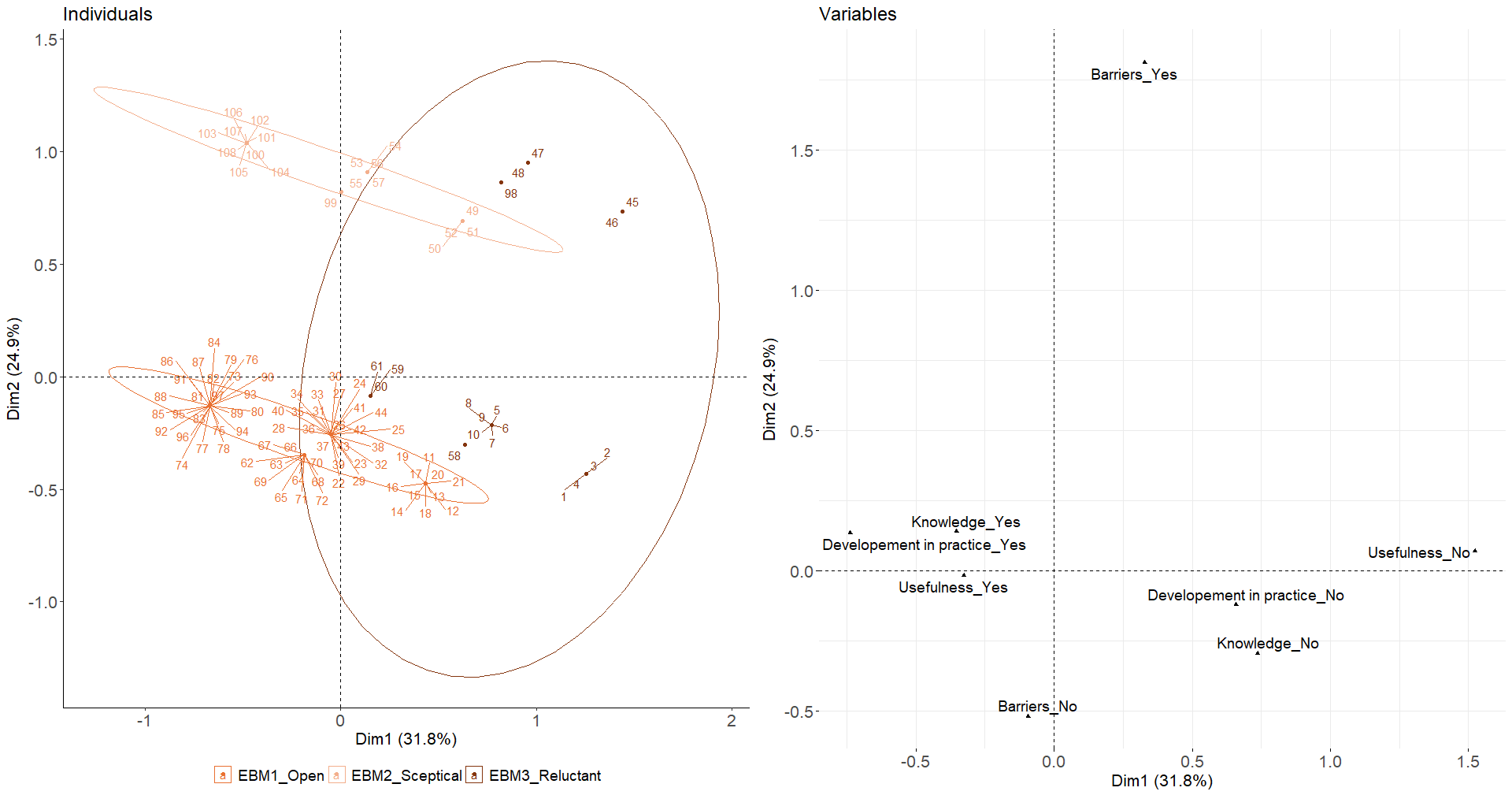


*Legend: MCA biplot illustrating the distribution of individuals and the contribution of categorical and continuous variables. The individuals are represented as points, while categorical variables are displayed as vectors. The plot shows the relationships between the first and second principal components, with continuous variables shown on the axes and categorical variables color-coded for clarity. The variance explained by each component is also indicated in the inset.*

**Appendix 5. Illustration of the three profiles of the swine veterinarians regarding PRRSV**


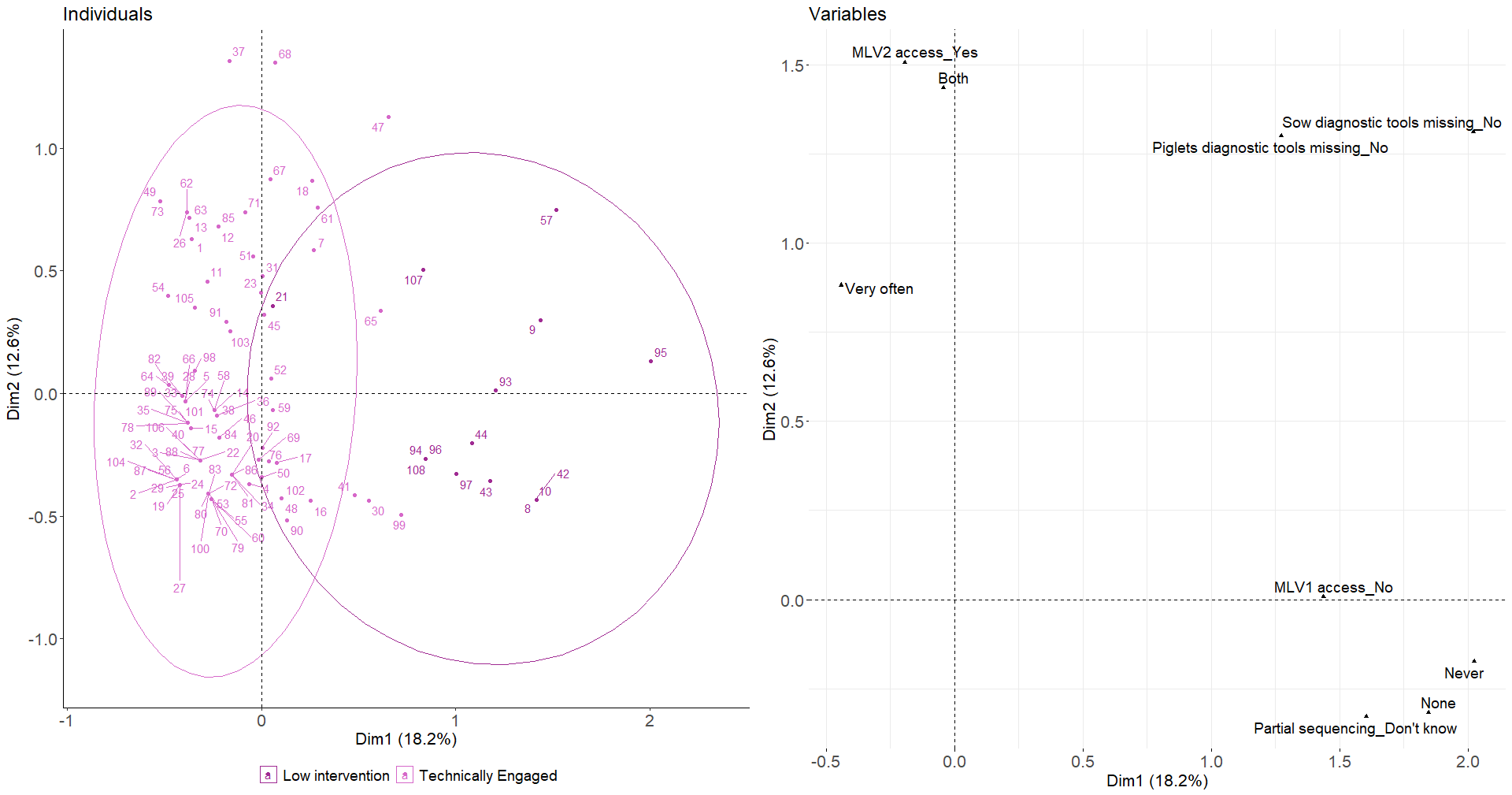


*Legend: MCA biplot illustrating the distribution of individuals and the contribution of categorical and continuous variables. The individuals are represented as points, while categorical variables are displayed as vectors. The plot shows the relationships between the first and second principal components, with continuous variables shown on the axes and categorical variables color-coded for clarity. The variance explained by each component is also indicated in the inset.*

**Appendix 6. Illustration of the three profiles of the swine veterinarians regarding their PRRSV stabilisation programs’ experiences**


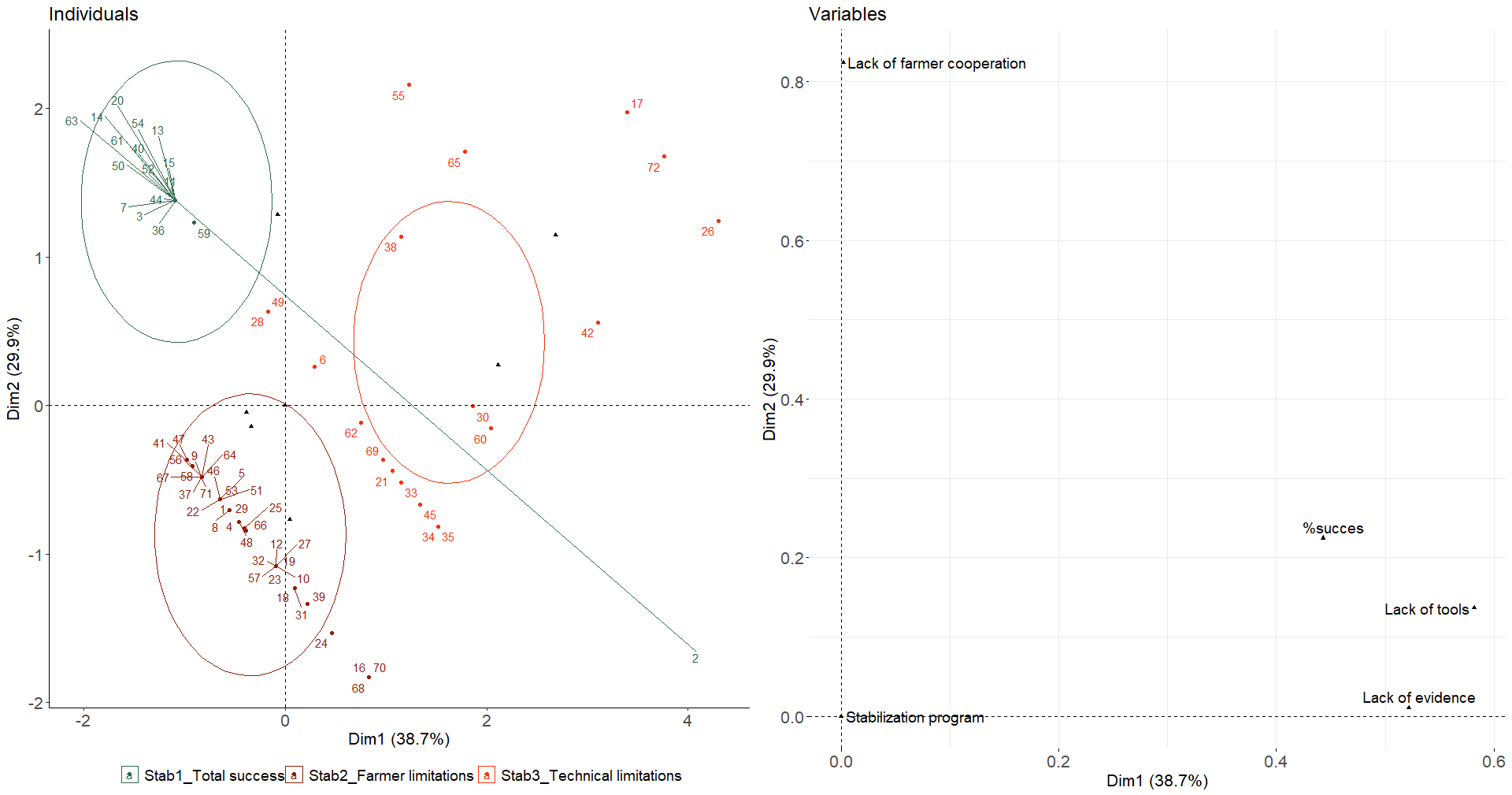


*Legend: FAMD biplot illustrating the distribution of individuals and the contribution of categorical and continuous variables. The individuals are represented as points, while categorical variables are displayed as vectors. The plot shows the relationships between the first and second principal components, with continuous variables shown on the axes and categorical variables color-coded for clarity. The variance explained by each component is also indicated in the inset.*
